# Intracranial artery stenosis magnetic resonance imaging aetiology and progression study: Rationale and design

**DOI:** 10.1002/brb3.1154

**Published:** 2018-11-19

**Authors:** Yongjun Han, Huiyu Qiao, Shuo Chen, Jing Jing, Yuesong Pan, Dongye Li, Yang Liu, Xia Meng, Yilong Wang, Xihai Zhao, ICASMAP investigators

**Affiliations:** ^1^ Center for Brain Disorders Research Beijing Institute of Brain Disorders Capital Medical University Beijing China; ^2^ Department of Biomedical Engineering Center for Biomedical Imaging Research Tsinghua University School of Medicine Beijing China; ^3^ Department of Neurology Beijing Tiantan hospital Capital Medical University Beijing China; ^4^ Department of Epidemiology and Health Statistics School of Public Health Capital Medical University Beijing China; ^5^ Department of Radiology Affiliated Hospital of Yangzhou University Yangzhou China; ^6^ Center of Stroke Beijing Institute for Brain Disorders Beijing China

**Keywords:** atherosclerosis, etiology, intracranial artery, magnetic resonance imaging, progression, stenosis

## Abstract

**Background:**

It has been shown that intracranial artery stenosis (ICAS) plays a key role in Chinese ischemic stroke or transient ischemic attack (TIA) patients. Many vascular diseases can lead to ICAS, such as atherosclerosis, dissection, vasculitis, moyamoya disease, and reversible cerebral vasoconstriction syndrome (RCVS). In addition, progression of intracranial atherosclerotic disease (ICAD) will increase the risk of ischemic cerebrovascular events. The ICASMAP study primarily aims to determine the etiology and disease distribution of ICAS using noninvasive magnetic resonance (MR) imaging and evaluate the rate for progression of ICAD in symptomatic population.

**Methods:**

The ICASMAP study is a prospective, observational, and multicenter study by recruiting 300 subjects (18–80 years old) with recent stroke or TIA (within 2 weeks after onset of symptoms) in China. All the subjects will undergo MR imaging examination including brain and intracranial artery MR imaging at baseline. In addition, the clinical risk factors will be collected and blood biomarkers will be tested. A subgroup of more than 200 subjects who were diagnosed with ICAD according to baseline MR imaging will be followed up for 2 years. During the follow‐up study, MR imaging examination will be performed at 12 and 24 months. The primary end point is presence of progression of intracranial artery atherosclerotic plaques.

**Conclusions:**

The ICASMAP study investigates the etiology of ICAS and progression of ICAD in Chinese stroke patients and may help to improve the precise diagnosis and intervention of ICAS and stroke prevention.

## 1 INTRODUCTION

Stroke is the second most common cause of death worldwide (Group GBDNDC, 2017; Truelsen et al., 2015) and has become the leading cause of death in China (Wang et al., 2017). Previous studies demonstrated that intracranial artery stenosis (ICAS) is significantly associated with ischemic stroke, particularly in Chinese population. A study (Wang et al., 2014) reported that 46.6% of Chinese patients with ischemic stroke had severe intracranial artery stenosis (stenosis > 50%). Although most of stenotic diseases in intracranial arteries are atherosclerotic (Hart et al., 2014), a substantial number of other vascular diseases, such as dissection, arteritis, moyamoya disease, and reversible cerebral vasoconstriction syndrome (RCVS), can also lead to intracranial artery luminal narrowing (Obusez et al., 2014; Sikkema et al., 2014; Yuan et al., 2015). Therefore, accurate diagnosis of the etiology of ICAS is important to personalize treatment strategies.

It has been shown that the progression of intracranial atherosclerotic disease (ICAD) will subsequently increase the risk of ischemic cerebrovascular events (Arenillas et al., 2001; Wong, Li, Lam, Chan, & Kay, 2002). Previous studies have shown that baseline symptomatic ICAD (Ryu et al., 2014), diabetes (Miyazawa, Akiyama, & Yamagata, 2007), smoking (Miyazawa et al., 2007), and treatment of statin (Kim, Kim, Kwon, Kim, & Kang, 2012) and cilostazol (Kwon et al., 2005) were associated with ICAS progression. Shimizu et al. (2013) summarized the inflammatory biomarkers associated with progression of ICAS, such as interleukin‐6, interleukin‐18, C‐reactive protein, intercellular adhesion molecule 1, and E‐selectin. However, the progression of ICAD may not be parallel to the changes of luminal stenosis. Currently, the risk factors for ICAD progression determined by metrics beyond luminal stenosis, such as plaque size and plaque compositions, remain unclear. Investigation of the risk factors of ICAD progression will be helpful for stabilizing ICAD and stroke prevention.

Magnetic resonance (MR) vessel wall imaging is capable of accurately evaluate vascular diseases that lead to ICAS according to the features of location, shape, signal pattern, remodeling, and contrast enhancement of lesions (Choi, Jung, & Lee, 2015; Mossa‐Basha et al., 2015). However, the results of recent studies on distribution of etiology of symptomatic ICAS are controversial. A MR vessel wall imaging study by Ahn et al. (2015) showed that moyamoya disease was the most dominant etiology of unilateral middle cerebral artery (MCA) stenosis in South Korean young adult patients with none or one atherosclerosis risk factor, followed by atherosclerosis, dissection, and vasculitis. However, a recent MR vessel wall imaging study reported that the most common etiology of ICAS in Chinese young patients with unilateral middle cerebral artery stenosis was ICAD (Xu et al., 2017). In addition, investigators have proved that MR vessel wall imaging is a reproducible technique (Qiao, Anwar et al., 2016; Qiao, Guallar et al., 2016), which can be reliably utilized to monitor the changes of ICAD during natural follow‐up or medical treatment.

In this article, we show the rationale and design of ICASMAP (Intracranial Artery Stenosis MR imaging: Aetiology and Progression) study. ICASMAP is a prospective, observational, multicenter study that aimed to investigate the etiology of symptomatic ICAS and the progression of ICAD using MR vessel wall imaging. The initiation of this study will improve the accuracy of diagnosis and intervention of ICAS and stroke prevention.

Therefore, the primary objectives of ICASMAP study are as follows: (a) to determine the etiology and disease distribution of ICAS using MR vessel wall imaging in symptomatic patients and (b) to evaluate the rate for progression of ICAD during 2‐year follow‐up. The secondary objective was to investigate risk factors for progression of ICAD.

## 2 METHODS

### 2.1 Study design and population

**Figure 1:**
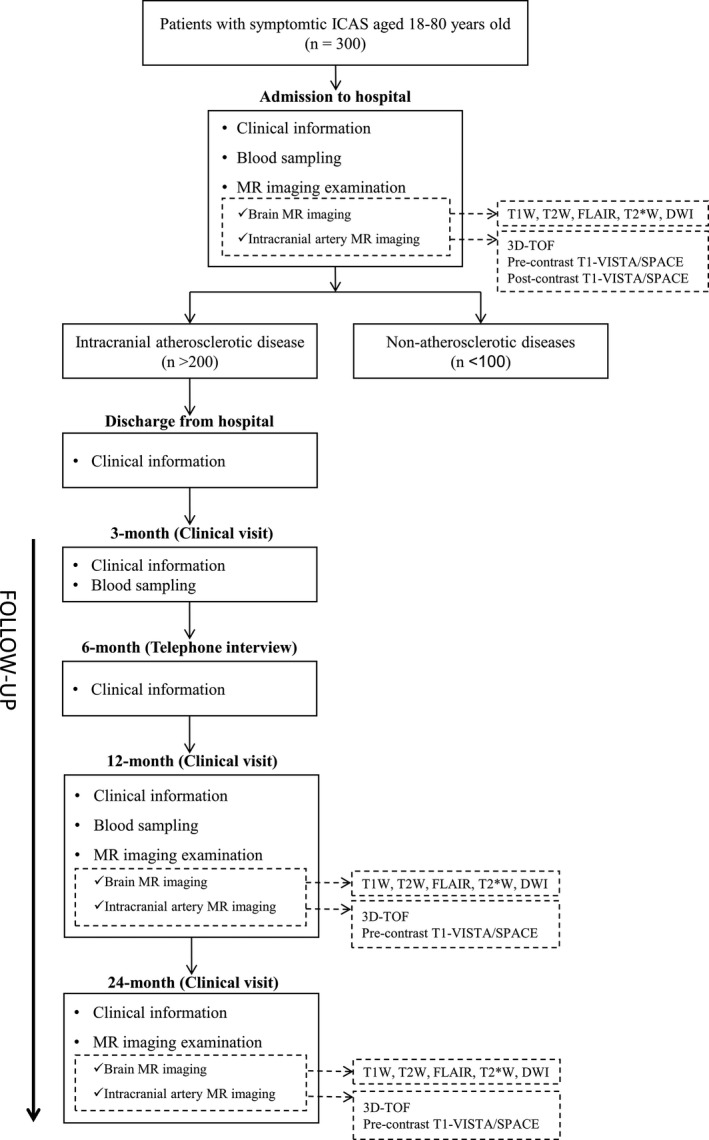
Flow chart for study procedures of ICASMAP. ICAS, intracranial artery stenosis; MR, magnetic resonance; T1W, T1‐weighted; T2W, T2‐weighted; FLAIR, T2‐fluid‐attenuated inversion recovery; T2*W, T2*‐weighted; DWI, diffusion‐weighted image; 3D‐TOF, three‐dimensional time‐of‐flight; T1‐VISTA, T1 volumetric isotropic turbo spin echo acquisition; T1‐SPACE, T1 sampling perfection with application‐optimized contrast using different flip angle evolutions

The ICASMAP study (NCT03417063) is a prospective, observational, and multicenter study. This study planned to recruit 300 patients (age range: 18–80 years old) who had recent ischemic stroke or transient ischemic attack (TIA; within 2 weeks after onset of symptoms) and ICAS (stenosis range from 30% to 99% that is the responsible lesion for symptoms) in at least one vascular bed determined by computed tomography angiography (CTA) or MR angiography. The ICAS lesions can be located in intracranial internal carotid artery, basilar artery, intracranial segment of vertebral artery (V4), M1 segment of middle cerebral artery, A1 segment of anterior cerebral artery, or P1 segment of posterior cerebral artery. The patients will be recruited from 18 different hospitals across Beijing–Tianjin–Hebei region in China. The exclusion criteria include the following: (a) severe carotid artery atherosclerotic disease (stenosis ≥ 70%); (b) cardiogenic thrombosis; (c) heart failure or respiratory failure; (d) renal dysfunction (serum creatinine ˃133 μmol/L); (e) serious disturbance of consciousness; (f) cerebral neoplasms; (g) intracranial hemorrhage; (h) claustrophobia; (i) contraindications to MRI; and (j) pregnant or plan to pregnant within recent 2 years. All subjects will undergo brain and intracranial artery MR imaging. For subjects who were diagnosed with ICAD (*n* > 200), the clinical follow‐up will be conducted at 3, 6, 12, and 24 months and the MR imaging follow‐up will be performed at 12 and 24 months, respectively. Previous studies reported that the progression rate of ICAS was 3.86% per year (Ryu et al., 2014) and the intra‐reader and inter‐scan variation in measuring maximum wall thickness of intracranial plaques was 6.67% (Zhang et al., 2018). Therefore, it might be reasonable to detect the 2 years’ progression of intracranial atherosclerosis disease (7.72%) which is greater than the variation of quantitative measurement by MRI. A flow chart of the study procedures is presented in Figure 1. The study protocol was approved by local Institutional Review Board, and all patients provide written informed consent.

### 2.2 Clinical data collection

Each patient will undergo clinical visit at admission to hospital, discharge from hospital, 3, 12, and 24 months and telephone interview at 6 months. During each visit, demographic and clinical information (age, gender, body mass index [BMI], smoking, diabetes, hypertension, hyperlipidemia, use of antihypertensive, hypoglycemic drugs, lipid‐lowering drugs, anticoagulants, and antiplatelet drugs) and clinical events (new stroke [ischemic and hemorrhage], TIA, cerebrovascular recanalization, or death) will be recorded. The information of the history of smoking, the recent smoking and former smoking is recorded. Diabetes is diagnosed by demonstrating any one of the following conditions: fasting blood sugar level ≥126 mg/dl; 2‐hr oral glucose tolerance test result ≥200 mg/dl; or hemoglobin A1c ≥6.5%. Hypertension is defined as diastolic blood pressure ≥90 mmHg or systolic blood pressure ≥140 mmHg. The levels of lipoprotein including high‐density lipoprotein, low‐density lipoprotein, total cholesterol and triglycerides are recorded. Hyperlipidemia means elevated concentrations of any or all of the following lipids in the plasma: low‐density lipoprotein >140 mg/dl; total cholesterol >200 mg/dl; or triglycerides >150 mg/dl.

### 2.3 Blood biomarker test

The blood draw will be conducted at median cubital vein at admission to hospital, 3 and 12 months. The blood biomarkers including fasting glucose, hemoglobin, cholesterol level, homocysteine, serum uric acid, high‐sensitivity C‐reactive protein, intercellular adhesion molecule 1, E‐selectin, matrix metallopeptidase 9, plasminogen activator inhibitor‐1, lipoprotein (a), interleukin‐6, interleukin‐18, macrophage chemoattractant protein‐1, oxidized low‐density lipoprotein, and lipoprotein‐associated phospholipase A2 will be tested.

### 2.4 MR imaging

Baseline MR examination includes brain MR imaging and intracranial artery high‐resolution MR vessel wall imaging. All MR examinations will be performed on 3.0T Philips or Siemens MR scanners with 8‐channel phase‐array head coil or 16‐channel neurovascular coil. Brain MR imaging was conducted using a standard protocol including T1‐weighted (T1W), T2‐weighted (T2W), T2‐fluid‐attenuated inversion recovery (FLAIR), T2*‐weighted (T2*W), and diffusion‐weighted image (DWI) sequences. The imaging parameters are detailed in Table 1. The intracranial artery MR vessel wall imaging protocol includes three‐dimensional (3D) time‐of‐flight (TOF) MR angiography and pre‐ and post‐contrast T1 volumetric isotropic turbo spin echo acquisition (VISTA) at Philips MR platform or T1 sampling perfection with application‐optimized contrast using different flip angle evolutions (SPACE) sequence at Siemens MR platform. The 3D TOF imaging sequence was acquired with the following parameters: turbo field echo/fast low angle shot sequence, repetition time 25/21 ms, echo time 3.5/3.6 ms, field of view 180 × 180/173 × 199 mm³, matrix 300 × 300/384 × 301, and thickness 0.6 mm. The VISTA/SPACE imaging sequence was acquired using the following parameters: fast spin echo sequence, repetition time 800/900 ms, echo time 19/24 ms, field of view 200 × 181 × 45/158 × 158 × 158 mm³, matrix 332 × 300 × 150/256 × 256 × 246, and thickness 0.6 mm. The intracranial artery MR imaging parameters are presented in Table 2. The post‐contrast MR vessel wall imaging will be performed at baseline by intravenous administration of gadolinium contrast agent (Magnevist, Bayer Schering Pharma AG, Berlin, Germany) with the dose of 0.1 mmol/Kg. The recruited patients who were diagnosed with atherosclerosis in intracranial arteries by baseline MR vessel wall imaging will be eligible for 2‐year follow‐up study using MR imaging. The MR vessel wall imaging in the follow‐up study will be performed at the same MR platform to the baseline at 12 and 24 months by acquiring pre‐contrast T1‐VISTA or T1‐SPACE imaging sequence with the same imaging parameters to the baseline.

**Table 1:** Brain MR imaging parameters

	T1W	T2W	T2‐FLAIR	DWI
Sequence	FFE^a^/FISP^b^	TSE	TSE	EPI
TR, ms	233	3,000	7,000	2,858
TE, ms	4.6	80	140	92
FOV, mm³	230 × 183 × 133	230 × 183 × 133	230 × 230 × 133	230 × 230 × 133
Matrix	400 × 255	400 × 255	256 × 195	128 × 126
Thickness, mm	5.5	5.5	5.5	5.5
Scan time	1′58″	1′48″	1′52″	34″

FFE, fast field echo; FISP, fast imaging with steady‐state precession; TSE, turbo spin echo; EPI, echo planar imaging; TR, repetition time; TE, echo time; FOV, field of view.

^a^The imaging sequence is from Philips MR platform.^b^The imaging sequence is from Siemens MR platform; All the imaging orientations are transverse.

**Table 2:** Intracranial artery MR vessel wall imaging parameters

	3D TOF	T1‐VISTA^a^/SPACE^b^
Sequence	TFE^a^/FLASH^b^	TSE
TR, ms	25/21	800/900
TE, ms	3.5/3.6	19/24
Echo train length	‐	30/27
FOV, mm³	180 × 180/173 × 199	200 × 181 × 45/158 × 158 × 158
Matrix	300 × 300/384 × 301	332 × 300 × 150/256 × 256 × 246
Thickness, mm	0.6	0.6
Scan time	5′39″/6′	7′01″/8′06″
Orientation	Axial	Axial/Coronal

3D TOF, three‐dimensional time‐of‐flight; FOV, field of view; FLASH, fast low angle shot; FSE, fast spin echo; T1‐VISTA, T1 volumetric isotropic turbo spin echo acquisition; T1‐SPACE, T1 sampling perfection with application‐optimized contrast using different flip angle evolutions; TE, echo time; TFE, turbo field echo; TR, repetition time.

^a^The imaging sequence is from Philips MR platform.^b^The imaging sequence is from Siemens MR platform.

### 2.5 MR image analysis

**Figure 2:**
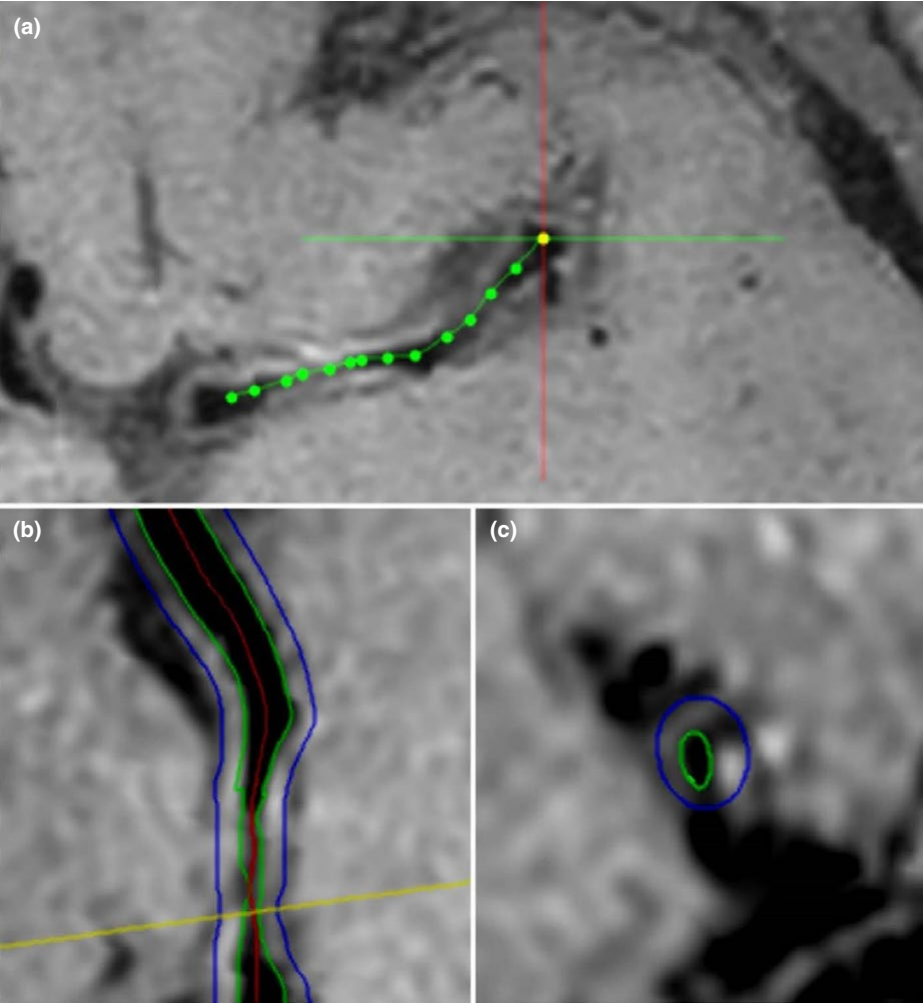
Work flow of the MR image analysis of intracranial artery using 3D‐CASCADE software. First step: outline of the center line of target segment of intracranial artery (a, M1 segment of middle cerebral artery); second step: automatic detection of the lumen and outer wall boundaries on the carved reconstructed image of target arterial segment (b); third step: manual adjustment of the lumen and outer wall boundaries on the axial image which is perpendicular to the center line (c)

All MR images will be transferred to the core lab of Center for Biomedical Imaging Research of Tsinghua University (Tsinghua University, Beijing, China) for centralized image review. The MR images will be interpreted by two experienced neuroradiologists blinded to clinical information and acquisition time point with consensus. The volume and location of acute cerebral infarcts on DWI and white matter lesions on FLAIR images (Liao et al., 1996) will be assessed. The MR vessel wall images will be reviewed using a custom‐designed software 3D‐CASCADE (Tsinghua University, Beijing, China). The lumen and outer wall boundaries were outlined automatically and adjusted manually. The lumen area, wall area, total vessel area, and maximum wall thickness for each stenotic lesion will be measured. The work flow of the MR image analysis of intracranial artery using 3D‐CASCADE software is shown in Figure 2. The luminal stenosis will be measured on the maximum intensity project of 3D TOF MRA images and MR vessel wall images using WASID criteria (Samuels, Joseph, Lynn, Smith, & Chimowitz, 2000) with the following grades: (a) 30%–50%; (b) 50%–70%; (c) 70%–99%; and (d) 100% (occlusion). The etiology of ICAS will be determined by experienced neuroradiologists and neurologists (>5 years’ experience) according to the characteristics of morphology, signal intensity, enhancement pattern, remodeling (Table 3; Choi et al., 2015), and clinical information. The enhancement pattern will be evaluated compared to the normal vessel wall using the following grades (van der Kolk et al., 2011): grade 0, no enhancement, the signal intensity of lesion is similar to or lower than that of normal wall; grade 1, the signal intensity of lesion is higher than that of normal wall but lower than that of pituitary stalk; and grade 2, the signal intensity of lesion is similar to or higher than that of pituitary stalk. The presence or absence of T1 hyperintensity will be identified which is defined as the signal intensity is 1.5 times higher than normal wall or brain tissue. The remodeling of lesion will be assessed with the published criteria: remodeling ratio (RR) which is calculated by the formula: RR = OWAlesion/(OWAreference + S*D). OWAlesion is then divided into the true outer wall area at the lesion site. OWAreference is defined as outer wall area at a reference point free of plaque within the vessel segment. *S* is the slope of the lumen tapering. *D* is the distance between the lesion and reference site. Positive, intermediate, and negative remodeling is defined as RR > 1.05, 0.95 ≤ RR ≤ 1.05, and RR < 0.95, respectively (Pasterkamp et al., 1997). For the MR vessel wall imaging data during follow‐up, the changes of luminal stenosis, wall thickness, T1 hyperintensity, contrast enhancement, and remodeling will be also measured.

**Table 3:** The imaging features of vascular diseases that lead to ICAS

	ICAD	Dissection	Arteritis	RCVS	MMD
Distribution	Any arteries	Any arteries (Intracranial: VA, BA)	The medium, small arteries of the meninges and cortex of the brain	Any arteries	Terminal ICA, Proximal MCA, Proximal ACA
Shape	Eccentric	Eccentric or combined	Concentric	Concentric	Concentric
Signal	Heterogenous	Hyperintensity (hematoma)	Homogenous	Homogenous	Homogenous
Enhancement	Dependent on vulnerability	Outer wall (±)	Diffuse enhancement	No or mild enhancement	Dependent on stage
Remodeling	Positive or negative	Unclear	Negative	Negative	Negative
Others	Intraplaque hemorrhage	Intimal flap, double lumen, aneurysmal dilatation	Exclusive diagnosis		Basal collaterals

ACA, anterior cerebral artery; BA, basilar artery; ICA, internal carotid artery; ICAS, intracranial artery stenosis; ICAD, intracranial atherosclerotic disease; MMD, moyamoya disease; MCA, middle cerebral artery; RCVS, reversible cerebral vasoconstriction syndrome; VA, vertebral artery.

### 2.6 End points

The presence of progression of intracranial artery atherosclerotic plaques is the primary end point. The progression of intracranial plaque is defined when it meets any of the following criteria at 1‐ or 2‐year follow‐up compared with baseline measurements: (a) the maximum wall thickness increased ≥10% compared with baseline; (b) the stenosis increased by one or more grades on TOF MRA or MR vessel wall imaging; (c) occurrence of T1 hyperintensity within plaque; or (d) the enhancement increased by one or more grades on post‐contrast T1 VISTA/SPACE images. Secondary end points include new ischemic stroke or TIA at any vascular territories, or all‐cause death.

### 2.7 Sample size estimation

According to previous studies, we assume about 20% of participants may have progression of intracranial atherosclerotic plaque in 2 years (Arenillas et al., 2008; Kwon et al., 2005; Ryu et al., 2014). Thus, 300 subjects are needed to estimate the progression rate of intracranial atherosclerotic plaque with a margin of error of 0.25× progression rate and a lost to follow‐up rate of 15%.

### 2.8 Reproducibility

Fifteen subjects were randomly selected for reproducibility study. One reader interpreted the intracranial MR images twice with time interval of 2 months for minimizing the memory bias. The second reader interpreted the intracranial MR images blinded to the review results of the first reader. During reproducibility study, readers will measure lumen area, wall area, total vessel area, maximum wall thickness, stenosis, remodeling, T1 hyperintensity, and contrast enhancement at intracranial artery lesions.

### 2.9 Statistical analysis plan

The continuous variables will be presented with mean ± standard deviation, and the categorical variables will be described as percentage. The prevalence of different vascular diseases, such as atherosclerosis, dissection, arteritis, moyamoya disease, and RCVS, that lead to ICAS in different age groups (<40, 40–50, 50–60, 60–70, and >70 years) will be calculated. The morphological measurements, T1 hyperintensity, enhancement patterns, and remodeling will be compared among different arterial diseases using One‐Way ANOVA or Mann–Whitney U test when appropriate. The progression rate of ICAD was calculated. Spearman correlation will be analyzed to determine the association of plaque features at baseline, clinical risk factors and blood biomarkers with progression of ICAD. Logistic regression model will be performed to calculate the odds ratio (OR) and corresponding confidence interval (CI) of plaque features at baseline, clinical risk factors and blood biomarkers in discriminating progression of ICAD before and after adjusting for confounding factors. Cox regression will be analyzed to calculate the hazard ratio and corresponding 95% CI of baseline intracranial artery vessel wall MR features in predicting for the cerebrovascular events. The intraclass correlation coefficient (ICC) and Kappa value were calculated for continuous and categorical variables in intra‐reader and inter‐reader reproducibility studies, respectively. All statistical analyses will be performed using SPSS 16.0 (SPSS Inc., Chicago, IL) and SAS (SAS Inc., North Carolina, NC).

## 3 DISCUSSION

The initiative of ICASMAP study utilizes MR vessel wall imaging to determine the etiology of ICAS and the rate of progression of ICAD during 2‐year follow‐up. This study may unveil the spectrum of diseases, including atherosclerosis, dissection, arteritis, moyamoya disease, and RCVS, that lead to ICAS in Chinese symptomatic patients. This study may also provide a clue to the ICAD progression associated risk factors which will be helpful for improving treatment strategies in clinical settings.

The spectrum of intracranial artery diseases that lead to ICAS has been studied in Asian young populations and targeted the middle cerebral artery. Ahn et al. (2015) studied 95 South Korean young patients (≤55 years old) with one or none atherosclerosis risk factor and unilateral severe stenosis in middle cerebral artery (stenosis > 50%) using high‐resolution MR vessel wall imaging. Investigators found that the most prevalent disease was moyamoya disease (30.5%), followed by atherosclerosis (27.4%), dissection (23.2%), and vasculitis (18.9%) in young adult patients with none or one atherosclerotic risk factor (Ahn et al., 2015). In contrast, Xu et al. (2017) recruited 122 Chinese young patients (from 18 to 45 years old) with unilateral middle cerebral artery stenosis to determine the etiology of ICAS using high‐resolution MR vessel wall imaging and found the most common etiology of ICAS was atherosclerosis (80.3%). The difference in prevalence of atherosclerotic disease between studies by Ahn et al. and Xu et al. may be due to the different definition of atherosclerosis and different profiles of study populations. The atherosclerosis was defined as eccentric wall lesion in Xu's study, whereas the lesion with eccentric and irregular wall thickening was defined as atherosclerotic disease in study by Ahn et al. The ICASMAP study will recruit symptomatic patients with wide range of age (from 18 to 80 years old) and larger sample size which may broaden the spectrum of etiology of ICAS in stroke patients.

MR vessel wall imaging has been widely used to differentiate the pathology of ICAS according to the imaging features of location, shape, signal pattern, remodeling, and contrast enhancement (Choi et al., 2015; Mossa‐Basha et al., 2015). Primarily, for assessing intracranial artery diseases, investigators utilized two‐dimensional (2D) imaging sequences which have the following disadvantages: (a) smaller coverage; (b) lower inter‐plane resolution; (c) insufficient blood suppression; and (d) long scan time. To overcome these technical limitations, three‐dimensional (3D) imaging techniques have been proposed and optimized for intracranial vessel wall imaging, particularly spin echo‐based sequence of VISTA (Qiao et al., 2011; Wang et al., 2016; Yang et al., 2016) or SPACE (Fan et al., 2017; Yang et al., 2017; Zhang et al., 2015). These 3D imaging sequences enable whole brain vessel wall imaging by providing large longitudinal coverage, isotropic spatial resolution, and excellent blood flow suppression. A recent consensus on intracranial MR vessel wall imaging recommended that T1‐weighted (or proton‐density‐weighted) vessel wall sequences before and after intravenous administration of gadolinium can be used to differentiate the etiology of ICAS (Mandell et al., 2017). In the ICASMAP study, pre‐ and post‐contrast enhanced 3D T1‐VISTA/SPACE sequences will be acquired to evaluate the intracranial artery wall in most of intracranial vascular beds. This study may provide more comprehensive view for lesion distribution in intracranial arteries.

The role of baseline intracranial artery plaque features in progression of ICAD remains unclear. Previous studies on relationship between baseline atherosclerosis and its progression are based on angiographic approaches. Mizukami, Shimizu, Maki, Shiraishi, and Hasegawa (2015) found that the global stenosis score, which is calculated by measuring the extent of middle cerebral arteries and the basilar artery, and the degree of carotid stenosis were significantly associated with progression of ICAS. Ryu et al. (2014) studied 102 subjects with ICAS and found that symptomatic ICAS subjects had greater risk of stenosis progression than asymptomatic ones. In carotid arteries, a number of studies have shown that presence of intraplaque hemorrhage (Sun et al., 2012; Takaya et al., 2005), maximum wall thickness (Xu et al., 2014), maximum lipid‐rich necrotic core percentage (Xu et al., 2014), and negative remodeling (Bianda et al., 2012) determined by MR vessel wall imaging are effective predictors for carotid plaque progression. However, there is no MR vessel wall imaging‐based evidence to show these relationships in ICAD. The ICASMAP study will provide a clue to the intracranial atherosclerotic plaque characteristics on MR vessel wall imaging at baseline that influence plaque progression.

It has been evidenced that some clinical risk factors and blood biomarkers were significantly associated with the progression of ICAD determined by angiography. A study by Miyazawa et al. (2007) showed that diabetes (OR, 6.771; *p* = 0.0004) and smoking (OR, 7.574; *p* = 0.0019) were correlated with the progression of ICAS. Shimizu et al. (2013) found that interleukin‐6 (HR, 1.215; 95% CI 1.002‐1.473) was a risk factor for the progression of ICAD in stroke patients. Another study by Arenillas et al. (2008) recruited 75 symptomatic ICAD patients and found that C‐reactive protein (CRP > 5.5 mg/L; HR, 5.4; 95% CI, 2.3–12.7; *p* = 0.0001) and plasminogen activator inhibitor‐1 (PAI‐1 > 23.1 ng/ml; HR, 2.4; 95% CI, 1.0–5.8; *p* = 0.05) were related to the progression of ICAD. In the clinical treatment aspect, investigators demonstrated that statin therapy may inhibit the progression of symptomatic ICAS in middle cerebral or/and basilar arteries (Kim et al., 2012). In addition, Kwon et al. (2005) provided evidence that cilostazol may prevent the progression of symptomatic ICAS. Since above studies are based on stenosis progression measured by angiographic approaches, the influence factors of plaque progression determined by MR vessel wall imaging remain unknown. The ICASMAP study will link potential influence factors to intracranial artery plaque progression assessed by MR vessel wall imaging and may help to improve the management of ICAD patients.

### 3.1 Limitations

Our study has several limitations. First, in this study, we recruited patients from Beijing–Tianjin–Hebei regions of China who represent urban residents. It will be interesting to assess the etiology of ICAS and progression of ICAD in rural regions in China in future studies. Second, this study focused on the patients who had 30%–99% stenosis. The etiology of intracranial artery with lower grade stenosis (<30% stenosis) is not studied. Third, this is MR vessel wall imaging study using different MR platforms (Philips and Siemens). The variability between Philips and Siemens MR scanner in measuring intracranial plaque features is unclear. Future studies focusing on the reproducibility among different MR platforms are warranted.

## 4 CONCLUSIONS

The ICASMAP study has been designed to investigate the etiology of ICAS and progression of ICAD in symptomatic adults using high‐resolution MR vessel wall imaging and to provide useful knowledge to improve stroke prevention.

## DISCLOSURE

All co‐authors claim no conflict of interest.
